# IgD Multiple Myeloma Paraproteinemia as a Cause of Myositis

**DOI:** 10.1155/2010/808474

**Published:** 2010-07-19

**Authors:** I. Colombo, M. E. Fruguglietti, L. Napoli, M. Sciacco, E. Tagliaferri, A. Della Volpe, V. Crugnola, N. Bresolin, M. Moggio, A. Prelle

**Affiliations:** ^1^Centro Dino Ferrari, Fondazione IRCCS Cá Granda Ospedale Maggiore Policlinico, 20122 Milano, Italy; ^2^Dipartimento di Neuroscienze, Azienda Ospedaliera Niguarda Cá Granda, 20122 Milano, Italy; ^3^Ematologia, Fondazione IRCCS Cá Granda Ospedale Maggiore Policlinico, 20122 Milano, Italy; ^4^Dipartimento di Neuroscienze, Azienda Ospedaliera Fatebenefratelli e Oftalmico, 20122 Milano, Italy

## Abstract

A 48-years old man was diagnosed an IgD-k multiple myeloma (MM) at age 38 years for which he successfully underwent chemotherapy and bone marrow transplant. He then developed a graft-versus-host disease (GVHD) whose manifestations included, three years later, a polymyositis, diagnosed at muscle biopsy and successfully treated with steroids. Few months after polymyositis remission, myeloma relapsed and the patient was treated with thalidomide for six years with good remission. Soon after thalidomide suspension, MM relapsed again and the patient came to our observation for a new onset of neuromuscular symptoms. He underwent both muscle and peripheral nerve biopsy to discriminate between myositis (paraproteinemia versus GVHD), amyloidosis, and thalidomide toxicity. The first muscle biopsy showed an inflammatory pattern with necrotic fibres, macrophagical invasion (CD68 positive), rare interstitial cellular infiltrates (CD8 positive and CD4 negative), widespread anti-HLA positivity and negative antiMAC. The second muscle biopsy showed the same inflammatory pattern plus an involvement of blood vessels. Direct immunofluorescence for IgD showed diffuse positivity along the sarcolemmal in both muscle biopsies. Sural nerve biopsy demonstrated both demyelinating and axonal aspects with no inflammatory infiltrates, but positivity for HLA and MAC. Congo Red was negative in both skeletal muscle and peripheral nerve.

IgD MM is a rare form of myeloma frequently associated with amyloidosis. Myositis is often described as expression of chronic GVHD, but not as a direct complication of MM. Second muscle biopsy revealed an inflammatory pattern with large amount of monoclonal proteins along the sarcolemma. Vessel inflammatory signs were also evident in peripheral nerve. This is the first case of concurrent polyneuropathy and myositis in IgD MM. We hypothesize that the neuro-myositis is caused by sarcolemmal deposits of IgD. The presence of these deposits correlates with high blood levels of monoclonal proteins.

 IgD myeloma is a rare entity, accounting for 1-2% of reported cases of multiple myeloma (MM) [1]. Association between demyelinating polyneuropathy or myositis and MM is known [2, 3]. Thalidomide, a treatment for refractory oncologic diseases, causes a predominantly sensory axonal neuropathy [4]. Myositis is a described complication of graft-versus-host disease (GVHD) presenting in a fashion similar to that of idiopathic myositis [5, 6]. Neuropathy may occur in a context of MM during systemic acquired amyloidosis or due to autoimmune process or cryoglobulinemia [7].

A 38-years-old man was diagnosed a IIA stadium IgD-k (MM). He immediately underwent chemotherapy (two DAV cycles and 4 ProMECE-CytaBOM cycles) and, one year later, bone marrow transplant (BMT). One month after BMT he developed a GVHD with skin and articular involvement. When he was 41 years old he developed muscular symptoms (myalgias and hyperCKemia). He underwent a skeletal muscle biopsy which led to a diagnosis of polymyositis: he was treated with steroids for some months and with azathioprine for three years with complete disease remission. 

At age of 42-years myeloma relapsed: the patient was treated with thalidomide for six years with good clinical remission, but progressively increasing levels of serum IgD were demonstrated.

 Six years later (at age of 48 years), the patient came to our observation for a new onset of neuromuscular symptoms: distal lower limb hypopallaesthesia, proximal limb hypotrophy, lower limb girdle weakness, hyperCKemia. Conduction studies examination showed changes suggestive of axonal polyneuropathy associated with a myopathic pattern on needle EMG investigation. 

 He underwent both muscle and peripheral nerve biopsy to discriminate between myositis (paraproteinemia versus GVHD), amyloidosis, and thalidomide toxicity. Conventional histological/histochemical studies along with immunohistochemistry for HLA, membrane attack complex (MAC), and IgD, were performed on both skeletal muscle biopsies and peripheral nerve cryostat sections. CD8, CD4, CD19, and CD68 immunohistochemistry was performed on skeletal muscle only. The first muscle biopsy showed an inflammatory pattern with necrotic fibres, macrophage invasion (CD68 positivity), rare interstitial cellular infiltrates, rare CD8 and absence of CD4 lymphocytes, widespread sarcolemmal HLA positivity, and no MAC detection in capillaries. The second muscle biopsy showed the same inflammatory pattern seen in the previous one plus an involvement of blood vessels (several perivascular cellular infiltrates in muscle with mild MAC positivity) and a slight increase of endomysial connective tissue. Sural nerve biopsy demonstrated both demyelinating and axonal aspects. No inflammatory infiltrates, but positive immunohistochemistry for HLA and MAC and dilated endoneurial capillaries were observed. 

 Congo Red was negative in both skeletal muscle and peripheral nerve.

Direct immunofluorescence for IgD was positive in both muscle biopsies: in the first one, IgD mainly accumulated along the sarcolemma; in the second one necrotic fibres and endomorphism connective were also strongly reactive. Conversely, nerve biopsy was IgD negative (see Figure 1). 

MM is associated with amyloidosis. In our case, no Congo Red positivity was observed in both muscle and nerve tissues. Cryoglobulins were absent in serum and autoimmune diseases were excluded by the absence of the most common antibodies. 

Myositis during chronic GVHD presents CD8-positive interstitial infiltrates with rare macrophages [6]. Our patient's biopsies showed a predominance of macrophages: these results do not favour GVHD diagnosis even if a systemic involvement was evident when the first myositis episode occured.

Thalidomide causes a neuropathy without inflammatory signs [4]. In our patient, nerve biopsy showed a mixed pattern with evidence of inflammation at immunohistochemistry. 

Kiprov and Miller [2] demonstrated IgG deposits along basement membrane and myelin sheath in case of myositis or demyelinating neuropathy during MM.

Given the presence of sarcolemmal and cytoplasmic deposits of IgD in both muscle biopsies, we hypothesize that myositis in our patient is paraproteinemia-correlated. The presence of IgD deposits correlates with high blood levels of monoclonal proteins at least in the second episode. 

Conversely, polyneuropathy could be caused by thalidomide, though inflammation cannot be excluded (HLA and MAC positivity) even if we did not observe IgD deposits. 

This is the first case of concurrent polyneuropathy and myositis in IgD MM. In our patient neuromuscular symptoms subsided after steroid therapy.

## Figures and Tables

**Figure 1:**
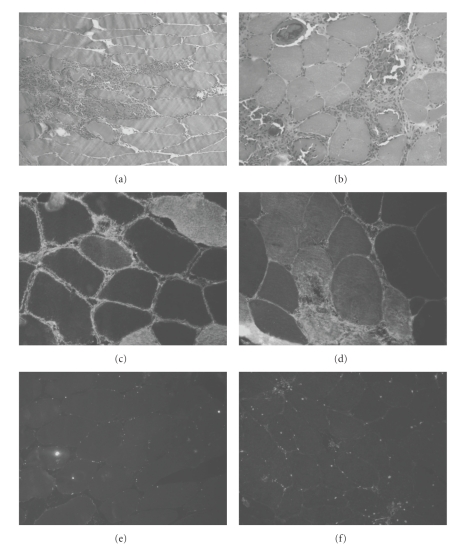
(a) GT 100X, first biopsy: presence of a large interstitial infiltrate. (b) HE 100X, second biopsy: perivascular infiltrates, mild increase of endomysial connective. (c) IgD immunofluorescence 250X, first biopsy: presence of immunoreactivity along the sarcolemma and in the connective tissue. (d) IgD immunofluorescence 250X, second biopsy: strong reactivity in necrotic fibres and endomysial connective. (e) IgD immunofluorescence in the negative control, 250X: aspecific signal. (f) IgD immunofluorescence in a case of vasculitis, 250X: aspecific signal.
